# Biocatalytic Synthesis of Poly(δ-Valerolactone) Using a Thermophilic Esterase from *Archaeoglobus fulgidus* as Catalyst

**DOI:** 10.3390/ijms131012232

**Published:** 2012-09-25

**Authors:** Hong Cao, Haobo Han, Guangquan Li, Jiebing Yang, Lingfei Zhang, Yan Yang, Xuedong Fang, Quanshun Li

**Affiliations:** 1 Key Laboratory for Molecular Enzymology and Engineering of Ministry of Education, College of Life Science, Jilin University, Changchun 130012, China; E-Mails: caohong1967@163.com (H.C.); hanhb1310@mails.jlu.edu.cn (H.H.); yangjiebing920320@yahoo.cn (J.Y.); fairy34100326@yahoo.cn (L.Z.); 2 Department of General Surgery, Second Hospital, Jilin University, Changchun 130041, China; E-Mail: guangquanli83@gmail.com

**Keywords:** thermophilic esterase, ring-opening polymerization, δ-valerolactone

## Abstract

The ring-opening polymerization of δ-valerolactone catalyzed by a thermophilic esterase from the archaeon *Archaeoglobus fulgidus* was successfully conducted in organic solvents. The effects of enzyme concentration, temperature, reaction time and reaction medium on monomer conversion and product molecular weight were systematically evaluated. Through the optimization of reaction conditions, poly(δ-valerolactone) was produced in 97% monomer conversion, with a number-average molecular weight of 2225 g/mol, in toluene at 70 °C for 72 h. This paper has produced a new biocatalyst for the synthesis of poly(δ-valerolactone), and also deeper insight has been gained into the mechanism of thermophilic esterase-catalyzed ring-opening polymerization.

## 1. Introduction

Poly(δ-valerolactone) (PVL) is an important aliphatic polyester due to its good biodegradability, biocompatibility, and permeability characteristics. Thus, it can be used as a hydrophobic block in amphiphilic block copolymers, which are suitable for constructing micellar delivery systems of hydrophobic antitumor drugs [1]. At present, PVL is mainly synthesized using organometallic catalysts, e.g., aluminium alkoxides, tin carboxylates and phosphido-diphosphine Group 3 complexes [2,3]. However, the trace metallic residues are not tolerated in biomedical applications due to their toxicity. In the past two decades, enzymatic ring-opening polymerization has been extensively developed [4–9], and has potential as a powerful route for synthesizing metal-free PVL or its copolymers under mild reaction conditions.

Compared with ɛ-caprolactone (the most widely investigated monomer), δ-valerolactone has attracted less attention in enzymatic ring-opening polymerization, mainly due to its relatively lower activity. Through the optimization of reaction conditions (enzyme origin, temperature, reaction medium and time), PVL was successfully synthesized in a biocatalytic route, with the highest monomer conversion and number-average molecular weight (*M*_n_) of 98% and 3200 g/mol, respectively [10]. Using ionic liquids as reaction medium, the ring-opening polymerization of δ-valerolactone with *Candida antarctica* lipase B (CALB) could also be realized, producing a product with a degree of polymerization of only 25 [11]. Similar to these results, previous work in our laboratory showed that a recombinant *Escherichia coli* whole-cell biocatalyst harboring the thermophilic lipase gene FN1333 could efficiently catalyze the ring-opening polymerization of δ-valerolactone, with monomer conversion of 97% and product *M*_n_ value of 1020 g/mol at 70 °C in toluene [12]. Michaelis-Menten kinetic analysis elucidated that due to the *cis* conformation of the ester bond of δ-valerolactone, CALB exhibited a similar affinity (*K*_m_ = 0.73 mol/L) to ɛ-caprolactone (*K*_m_ = 0.72 mol/L); nevertheless, the *k*_cat_ value for the CALB-catalyzed ring-opening polymerization of δ-valerolactone (*k*_cat_ = 35.2 s^−1^) was obviously lower than that of ɛ-caprolactone (*k*_cat_ = 72.9 s^−1^) [13–15]. Meanwhile, the monomer δ-valerolactone showed a much higher hydrolysis rate, almost 21-fold higher than ɛ-caprolactone [13], which was unfavorable for the enzymatic ring-opening polymerization. Thus, the monomer δ-valerolactone has relatively lower activity in enzymatic polymerization than ɛ-caprolactone, and is hard to polymerize enzymatically in a high yield with the product molecular weight. Potential strategies to solve this problem are to explore novel enzymes with high catalytic activity and stability from extremophiles, or to construct tailor-made biocatalysts *via* protein engineering techniques.

Thermophilic esterase from the hyperthermophilic archaeon *Archaeoglobus fulgidus* (AFEST) has been cloned, over-expressed in *E. coli* and well characterized with respect to substrate specificity, crystal structure and catalytic mechanism [16–19]. The enzyme was classified as a member of a hormone-sensitive lipase group of the esterase/lipase family, with an optimal temperature of 80 °C [17,18]. It could catalyze the hydrolysis of a broad range of esters, including *p*-nitrophenyl esters, vinyl esters, and triacylglycerols with a short chain, and it exhibited the highest activity towards *p*-nitrophenyl hexanoate among the *p*-nitrophenyl esters tested [17]. Previous work in our laboratory demonstrated that it could successfully catalyze the ring-opening polymerization of ɛ-caprolactone, with 100% monomer conversion and product *M*_n_ of 1400 g/mol in toluene at 80 °C for 48 h [20,21].

In the present study, ring-opening polymerization of δ-valerolactone was conducted using the thermophilic esterase AFEST as catalyst. Systematical optimization of the reaction conditions was performed to obtain high monomer conversion and product molecular weight, and thus was helpful for gaining deeper insight into the mechanism underlying the effects of the reaction conditions.

## 2. Results and Discussion

### 2.1. Purification of the Recombinant Enzyme AFEST

**Figure 1:**
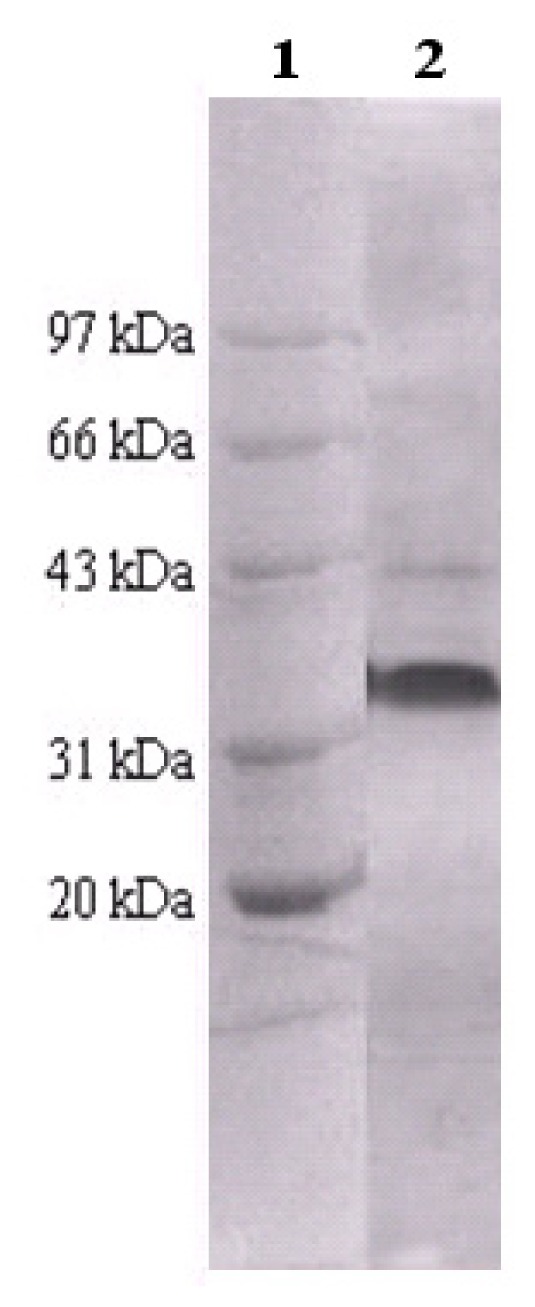
SDS-PAGE analysis of the recombinant enzyme AFEST. Lane 1: protein marker; Lane 2: the purified recombinant enzyme AFEST. Protein standards employed were: rabbit phosphorylase b (97 kDa), bovine serum albumin (66 kDa), rabbit actin (43 kDa), bovine carbonic anhydrase (31 kDa), and trypsin inhibitor (20 kDa).

The recombinant *E. coli* BL21 strain was first cultured and the cells were harvested through centrifugation. Afterwards, through ultrasonic cell disintegration and unique thermal precipitation strategy [22], the recombinant enzyme AFEST was considerably purified. Finally, ultrafiltration and lyophilization were employed to remove the fractions of low molecular weight and obtain the enzyme preparation, respectively. The SDS-PAGE analysis showed a major band at 35.5 kDa, which corresponded to the enzyme AFEST, and the purity was more than 92% (Figure 1). The lyophilized AFEST, with a specific activity of 168 U/mg towards *p*-nitrophenyl caprylate at 80 °C [20], was then used as catalyst in the ring-opening polymerization of δ-valerolactone.

**Figure 2:**
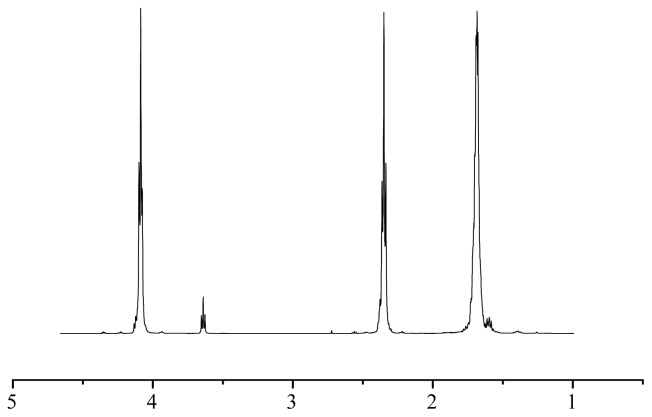
^1^H NMR spectrum of the product synthesized by AFEST-catalyzed ring-opening polymerization of δ-valerolactone in toluene at 70 °C for 72 h.

The ring-opening polymerization of δ-valerolactone was first performed using 30 mg AFEST in toluene at 70 °C for 72 h, and the product obtained was characterized by ^1^H NMR. As shown in Figure 2, the spectrum confirmed the structure of PVL as follows: 1.68 (m, –COCH_2_CH_2_CH_2_CH_2_O–), 2.35 (t, –COCH_2_–), 3.64 (t, –CH_2_OH end group), 4.09 (t, –CH_2_O–). Through the calculation of peak areas at 3.64 and 4.09 ppm ((Area_4.09_/Area_3.64_ + 1) × 100), the *M*_n_ value of the product was ca. 1540 g/mol. Compared with the conventional chemical route (e.g., PVL with molecular weight of 21,200 g/mol was obtained using phosphido-diphosphine Group 3 complexes [3]), the synthesized polymers in the present research are of low molecular weight, and might be used as the soft segments in thermoplastic elastomers, or carriers for controlled drug delivery and release.

### 2.2. Effect of Enzyme Concentration

**Figure 3:**
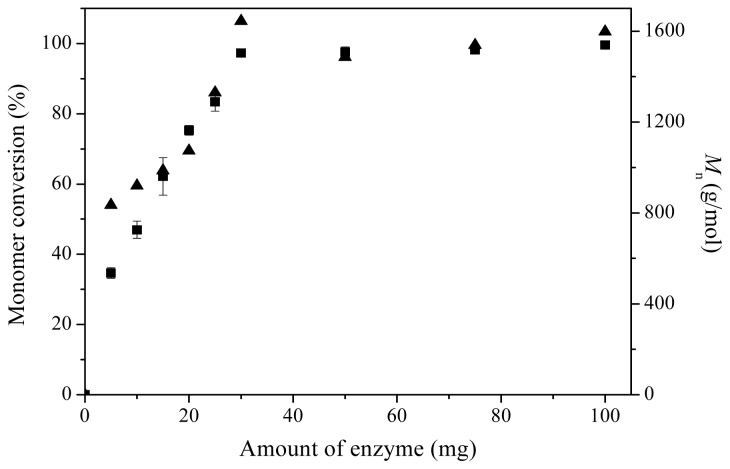
Monomer conversion (■) and *M*_n_ (▲) as a function of amount of enzyme for AFEST-catalyzed ring-opening polymerization of δ-valerolactone. The reactions were conducted in toluene at 80 °C for 72 h.

In the lipase-catalyzed polyester synthesis, enzyme concentration played an important role in both monomer conversion and product molecular weight [23]. The effect of enzyme concentration was investigated using different amounts of enzyme AFEST, 200 μL δ-valerolactone and 600 μL toluene at 80 °C for 72 h. Monomer conversion and *M*_n_ as a function of the amount of enzyme are shown in Figure 3. Obviously, increasing the enzyme concentration should be favorable for increasing monomer conversion, and when the amount of enzyme exceeded 30 mg, the monomers were completely converted (>97% monomer conversion). Similar to monomer conversion, *M*_n_ values also exhibited a tendency to increase at relatively low amounts of enzyme (<30 mg), and at large amounts of enzyme (>30 mg), *M*_n_ values remained constant (1490–1650 g/mol). However, in the AFEST-catalyzed ring-opening polymerization of ɛ-caprolatone, *M*_n_ values were almost independent of the monomer conversions, and remained constant in the range of 30–100 mg amount of enzyme [20]. This phenomenon was probably caused by the fact that large amounts of insoluble enzyme in the reaction system could lead to mass transfer limitation, and thus in the subsequent research, 30 mg AFEST was selected as the optimal amount of enzyme.

### 2.3. Effect of Temperature

**Figure 4:**
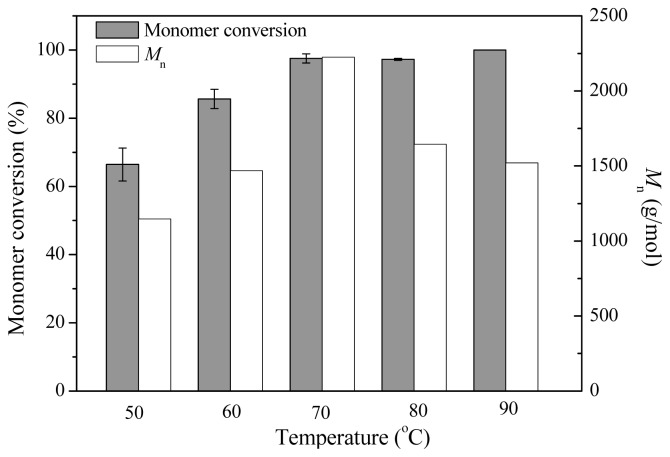
Effect of temperature on monomer conversion and product molecular weight *M*_n_. The reactions were carried out using 200 μL δ-valerolactone and 600 μL toluene at different temperatures in toluene for 72 h.

Effect of temperature on monomer conversion and product molecular weight was investigated at different temperatures in toluene for 72 h. As shown in Figure 4, high monomer conversions were achieved at high temperatures, and in the range of 70–90 °C, the monomers were almost completely consumed, with monomer conversion values of higher than 97%. For the product *M*_n_ values, reaction at 70 °C exhibited the highest value of 2225 g/mol, which was higher than that calculated through ^1^H NMR (1540 g/mol) as described above. In the AFEST-catalyzed poly(ɛ-caprolatone) synthesis [20], high temperature was favorable for the product molecular weight; and thus the results in the present study are different, but it is a common phenomenon, mainly due to the different binding affinity of AFEST towards different monomers. With regard to monomer conversion and *M*_n_ values, 70 °C was employed as the optimal reaction temperature.

### 2.4. Effect of Reaction Time

**Figure 5:**
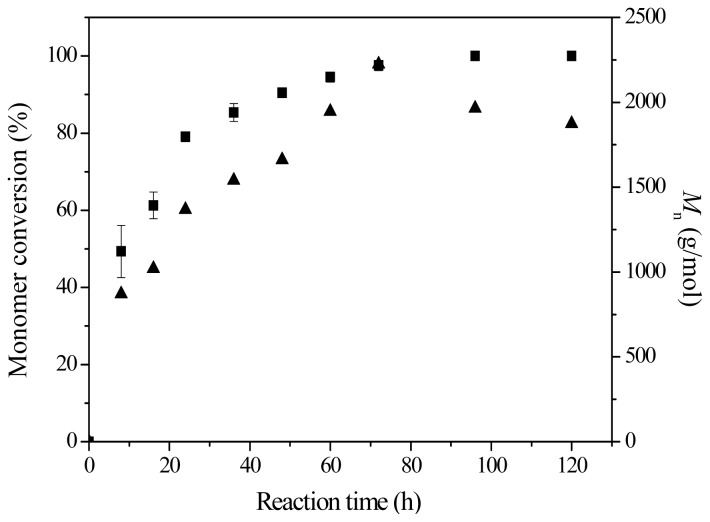
Monomer conversion (■) and *M*_n_ (▲) as a function of reaction time for AFEST-catalyzed ring-opening polymerization of δ-valerolactone. The reactions were conducted in toluene at 70 °C for different reaction times.

Time-course studies of AFEST-catalyzed ring-opening polymerization of δ-valerolactone were performed in toluene at 70 °C for different reaction times. Figure 5 shows the corresponding plots of monomer conversion and *M*_n_ vs. reaction time. During the polymerization before 72 h, the monomer conversion and *M*_n_ increased with reaction time. After 72 h, monomer conversion values could still increase from 97% (72 h) to 100% (96 and 120 h). However, the *M*_n_ values exhibited an obvious tendency to decrease after 72 h. In enzymatic ring-opening polymerization, enzymatic hydrolysis and transesterification are also involved in the reaction system [24], and thus we can conclude that before 72 h, rapid chain initiation and propagation could lead to the increasing monomer conversion and *M*_n_ values, and after 72 h, hydrolysis and transesterification of the formed polyester chains play important roles in the reaction system, which is probably the main reason for the decreasing *M*_n_ values. Interestingly, higher PDI values were also obtained in hydrophobic solvents. In addition to catalyzing polymerization, lipases and esterases can also cleave the ester bonds of polyesters, thus catalyzing the degradation, inter-transesterification and intra-transesterification of the polymer chain [24]. The use of hydrophobic solvents results in an improved degree of polymerization, but might also lead to increased rates of hydrolysis and transesterification; this in turn results in a polymer with a broader molecular weight distribution.

### 2.5. Effect of Reaction Medium

The nature of solvents employed plays a crucial role in determining the stability of the biocatalyst and in the partitioning of substrates and products between the solvent and the biocatalyst in non-aqueous biocatalytic systems [25]. The effect of organic solvents with different Log *P* values on monomer conversion and *M*_n_ at 70 °C for 72 h is summarized in Table 1 (Log *P* is the octanol/water partition coefficient value [26]). Similar to AFEST-catalyzed ring-opening polymerization of ɛ-caprolactone [20], high monomer conversion (>97%) and *M*_n_ values (1860–2225 g/mol) were achieved in hydrophobic solvents (toluene, cyclohexane and n-hexane), which could be ascribed to the deactivation of enzyme by the relatively hydrophilic solvents, since these solvents might disrupt the functional structure or strip off the essential water layer from the enzyme [27,28]. In addition, enzymatic ring-opening polymerization of δ-valerolatone could also proceed in a solvent-free system, with monomer conversion and *M*_n_ values of 94% and 1620 g/mol, respectively. In all, toluene was selected as the optimal reaction medium in the present research.

## 3. Experimental Section

### 3.1. Materials

Recombinant *E. coli* BL21 harboring the thermophilic esterase gene AF1716 from *A. fulgidus* was kindly provided by Dr. Giuseppe Manco (Istituto di Biochimica delle Proteine, Italy). Yeast extract and tryptone was purchased from Oxoid Ltd (Basingstoke, UK). Isopropyl β-d-thiogalactopyranoside (IPTG), ampicillin and *p*-nitrophenyl caprylate were purchased from Sigma (Shanghai, China). δ-Valerolactone was purchased from Sigma (Shanghai, China) in the highest available purity and used as received. Molecular sieves (4 Å) were purchased from Tianjin Chemical Co. (Tianjin, China), and heated at 500 °C for 3 h. Organic solvents of analytical grade were purchased from Beijing Chemical Co. (Beijing, China), and dried over 4 Å molecular sieves before use. All other reagents were of the highest reagent grade commercially available, and used without further purification.

### 3.2. Purification of the Recombinant Enzyme AFEST

The recombinant *E. coli* BL21 strain was cultured in 2YT medium (1% yeast extract, 1.6% tryptone, and 0.5% NaCl) containing ampicillin (100 μg/mL) at 37 °C. When the optical density at 600 nm of the culture reached a value of 1.8, the induction was performed by adding IPTG at a final concentration of 1 mM and shaking for an additional 3 h at 37 °C. The cells were harvested by centrifugation at 8000 rpm for 15 min, and washed with 50 mM phosphate buffer (pH 8.0). The harvested cells were frozen and thawed three times, and then suspended in 50 mM phosphate buffer (pH 8.0) at a ratio of 1:6 (*w*/*v*). After ultrasonic cell disintegration and incubation at 80 °C for 30 min, the suspension was then centrifuged at 8000 rpm for 15 min. The solution was collected, ultrafiltrated under a 10 kDa membrane and lyophilized to obtain the enzyme preparation.

The esterase activity was monitored by measuring the amount of *p*-nitrophenyl from the hydrolysis of *p*-nitrophenyl caprylate at 80 °C [29]. One unit of enzymatic activity was defined as the amount of protein releasing 1 μmol *p*-nitrophenyl from *p*-nitrophenyl caprylate in one minute.

### 3.3. Enzymatic Ring-Opening Polymerization of δ-Valerolactone

The thermophilic esterase AFEST was dried in a desiccator overnight, and then transferred to a dried screw-capped vial containing 200 μL δ-valerolactone and 600 μL organic solvent (no solvent addition for solvent-free system). Ethylbenzene (50 μL) was added as an internal standard for the quantification of monomer conversion by gas chromatography (GC). The vial was sealed and then placed into a thermostatic reactor with stirring (180 rpm) at the appropriate temperature. After the reaction, 5 mL dichloromethane was added, and the enzyme was removed by filtration. The enzyme was washed three times with dichloromethane, and the filtrate was collected and evaporated under reduced pressure to remove dichloromethane. Afterwards, the viscous sample was precipitated in methanol at −20 °C, and the cloudy solution was centrifuged (8000 rpm, 15 min, 4 °C). The white precipitate was then collected, dried in a vacuum oven, and then characterized by ^1^H NMR.

### 3.4. Structural Characterization of PVL

The structure of the polymer was characterized by ^1^H NMR. The ^1^H NMR spectrum was recorded in chloroform-d (CDCl_3_) on an AVANCE DMX 500 spectrometer at 500 MHz. The chemical shifts for ^1^H NMR spectra were referenced relative to tetramethylsilane (TMS, 0.00 ppm).

### 3.5. Determination of Monomer Conversion

Monomer conversion values were determined by GC using a Shimadzu 2014 gas chromatograph (Shanghai, China) equipped with an Rtx-1 capillary column (30 m × 0.25 mm × 0.25 μm) and a hydrogen flame ionization detector. Nitrogen was used as the carrier gas. The temperatures of injection pool and detector were set as 200 °C and 240 °C, respectively. The column temperature was held at 70 °C for 2 min, and then programmed to rise at 10 °C/min to a final temperature of 140 °C, which was then maintained for 2 min. The injection volume was 1.0 μL.

### 3.6. Determination of Molecular Weight and Polydispersity Index (PDI)

The *M*_n_, weight-average molecular weight (*M*_w_) and PDI (*M*_w_/*M*_n_) values of products were determined by gel permeation chromatography (GPC). Analyses were carried out using a Shimadzu HPLC system (Shanghai, China) equipped with a refractive index detector and Shim-pack GPC-804 and GPC-8025 ultrastyragel columns in series. Tetrahydrofuran was used as the eluent with a flow rate of 1.0 mL/min at 40 °C. The sample concentration and injection volume were 0.3% (*w*/*v*) and 20 μL, respectively. The GPC system was calibrated with polystyrene standards of narrow molecular weight distribution.

## 4. Conclusions

In this paper, PVL was successfully synthesized using a thermophilic esterase AFEST from the archaeon *A. fulgidus* as catalyst. The synthesized PVL was of low molecular weight and narrow molecular weight distribution, and is expected to be widely used as the soft block of thermoplastic elastomers, or carriers for controlled drug delivery and release. This work provided a new green and sustainable route for polyester synthesis, and was also helpful in broadening the application scope of thermophilic esterases and facilitating insight into the mechanism of thermophilic esterase-catalyzed ring-opening polymerization.

## Figures and Tables

**Table 1:** Effects of organic solvents on monomer conversion and product molecular weight *M*_n_. The reactions were conducted using 200 μL δ-valerolactone and 600 μL toluene at 70 °C for 72 h in different organic solvents.

Solvent	Log *P*	Monomer conversion (%)	*M*_n_ (g/mol)	PDI
1,4-Dioxane	−1.10	86	860	1.09
Acetone	−0.23	87	850	1.12
Tetrahydrofuran	0.49	93	910	1.10
Dichloromethane	0.93	72	860	1.14
Chloroform	2.00	91	960	1.15
Toluene	2.50	97	2225	1.37
Cyclohexane	3.09	100	1860	1.28
n-Hexane	3.50	100	1970	1.29
Solvent-free	-	94	1620	1.26
